# Transformation of the matrix structure of shrimp shells during bacterial deproteination and demineralization

**DOI:** 10.1186/1475-2859-12-90

**Published:** 2013-10-05

**Authors:** Youmei Xu, Mini Bajaj, Reinhard Schneider, Stephan L Grage, Anne S Ulrich, Josef Winter, Claudia Gallert

**Affiliations:** 1 Institute of Biology for Engineers and Biotechnology of Wastewater, Karlsruhe Institute of Technology, Am Fasanengarten, Karlsruhe 76131, Germany; 2 Laboratory for Electron Microscopy, Karlsruhe Institute of Technology, Engesserstrasse 7, Karlsruhe 76131, Germany; 3 Institute for Biological Interfaces (IBG-2), Karlsruhe Institute of Technology, P.O.B. 3640, Karlsruhe 76021, Germany; 4 Faculty of Technology, Department of Microbiology/Biotechnology, Hochschule Emden-Leer, Constantiaplatz 4, Emden 26732, Germany

**Keywords:** *Penaeus monodon*, Shrimp shells, Proteolytic enrichment cultures, Lactic acid bacteria (LAB), ^13^C solid state NMR, Electron microscopy, Chitin structure, Viscosity

## Abstract

**Background:**

After cellulose and starch, chitin is the third-most abundant biopolymer on earth. Chitin or its deacetylated derivative chitosan is a valuable product with a number of applications. It is one of the main components of shrimp shells, a waste product of the fish industry. To obtain chitin from *Penaeus monodon,* wet and dried shrimp shells were deproteinated with two specifically enriched proteolytic cultures M1 and M2 and decalcified by *in-situ* lactic acid forming microorganisms. The viscosity of biologically processed chitin was compared with chemically processed chitin. The former was further investigated for purity, structure and elemental composition by several microscopic techniques and ^13^C solid state NMR spectroscopy.

**Results:**

About 95% of the protein of wet shrimp shells was removed by proteolytic enrichment culture M2 in 68 h. Subsequent decalcification by lactic acid bacteria (LAB) took 48 h. Deproteination of the same amount of dried shrimps that contained a 3 × higher solid content by the same culture was a little bit faster and was finished after 140 h. The viscosity of chitin was in the order of chemically processed chitin > bioprocessed chitin > commercially available chitin. Results revealed changes in fine structure and chemical composition of the epi-, exo- and endocuticle of chitin from shrimp shells during microbial deproteination and demineralization. From transmission electron microscopy (TEM) overlays and electron energy loss spectroscopy (EELS) analysis, it was found that most protein was present in the exocuticle, whereas most chitin was present in the endocuticle. The calcium content was higher in the endocuticle than in the exocuticle.^13^C solid state NMR spectra of different chitin confirmed < 3% impurities in the final product.

**Conclusions:**

Bioprocessing of shrimp shell waste resulted in a chitin with high purity. Its viscosity was higher than that of commercially available chitin but lower than that of chemically prepared chitin in our lab. Nevertheless, the biologically processed chitin is a promising alternative for less viscous commercially available chitin. Highly viscous chitin could be generated by our chemical method. Comprehensive structural analyses revealed the distribution of the protein and Ca matrix within the shrimp shell cuticle which might be helpful in developing shrimp waste processing techniques.

## Background

Besides cellulose and starch chitin is one of the most common biopolymers on earth. Chitin and its de-acetylated derivative chitosan have numerous applications in medicine, agriculture, biotechnology, cosmetics and in pharmaceutical, food, paper and textile industries [1,2]. It is estimated that annually 10^6^ - 10^7^ tons of chitin are produced in nature [3]. The rapid increase of the human population on earth and the resulting rising demand for raw materials requires the recycling of biological waste fractions to upgrade the major components, such as lignin, starch, cellulose or chitin. Chitin for example forms the outer skeleton of arthropods and is the main component of the cell walls of fungi. Unlike many synthetic polymers chitin is a sustainable biodegradable natural biopolymer. From purified and partially de-acetylated chitin, several chitin- or chitosan products are already on the market and many more applications are under consideration [4]. If purified from crustaceans or fungi, highly polymeric chitin or chitosan would be available in sufficient amounts for future uses.

Shrimp shells are a waste of the shellfish processing industry. Globally about 6.1 million tons of crustaceans were captured by trawlers from marine waters or harvested from inland waters in 2010 [5]. Out of total crustaceans, the annual worldwide production of *Paneaus monodon* shrimp species was 209 662 tons. In Indonesia about 48% of the capture is for export [5]. Since, generally not the whole animal as such but only the shrimp meat is exported, large quantities of shell waste (40-66% of shrimp weight) are generated, which cause environmental problems as no controlled disposal is available. Shrimp shells are burned, left for rotting on land, dried for chicken feed or simply discharged back into the sea. To solve the problem of disposal while generating value added products adhesive protein must be separated from the shells and the chitin must be decalcified and deacetylated to chitosan. Bacterial mediated digestion of protein and decalcification is an appropriate method to obtain not only chitin but also the digestion liquid which contains the valuable protein fraction, astaxanthin and solubilised calcium ions and might be used as animal feed supplements. However, chemical methods are still preferred because of their short processing time.

The ease of chitin extraction depends on the structure of the raw material and the location of chitin within shrimp shells. Shrimp shells consist of three outer calcified layers i.e. epi-, exo- and endocuticles and an inner membrane. According to Roer and Dillaman [6] and to several references mentioned within their paper, the mineralic incrustation exists in the form of calcite crystals and amorphous calcium carbonate. In the epicuticle, which is the outermost and the thinnest layer of the epidermis, minerals are present as spherulitic calcite islands, surrounded by a lipid-protein matrix. It is bi-laminar, traversed by the basal layer of mineral-filled channels at right angles. Located beneath the epicuticle is the exocuticle, consisting of chitin-protein fibres embedded with mineralic crystals, stacked in layers of continuously changing orientation. This continuously changing orientation of chitin-protein stripes occurs also in the endocuticle, which is the thickest and the most calcified layer of the shrimp shells and located beneath the exocuticle. The total organic material e.g. of shells of *Cancer pagurus* comprises up to 73% chitin by weight, making crustacean waste a valuable raw material for chitin production.

In this study, biological chitin purification from *Penaeus monodon* shrimp shells of Indonesia was carried out and its viscosity was compared with chemically extracted chitin. For deproteination, autochthonic protease secreting bacteria were enriched and for decalcification, lactic acid bacteria (LAB) from yoghurt were used. In order to evaluate the different states of deproteination and demineralization and to correlate these with the shrimp-shell structure, several imaging techniques, including light microscopy (LM) as well as scanning electron microscopy (SEM) and transmission electron microscopy (TEM) were applied. In addition, TEM was combined with electron energy loss spectroscopy (EELS) and energy-filtered TEM (EFTEM) for chemical analyses and two-dimensional imaging of the elemental distribution. These correlated imaging and analytical investigations including nuclear magnetic resonance (NMR) revealed the exact location of protein, chitin and calcium within the different layers of the *Penaeus monodon* cuticle before and after biological treatment steps for chitin purification. Microscopic and physical/chemical analyses might be essential tools to evaluate the effect of single treatment steps of shrimp waste shells for development of alternative ways for chitin production.

## Results and discussion

### Deproteination of wet and dried shrimp shells with enrichment cultures M1 and M2

**Figure 1:**
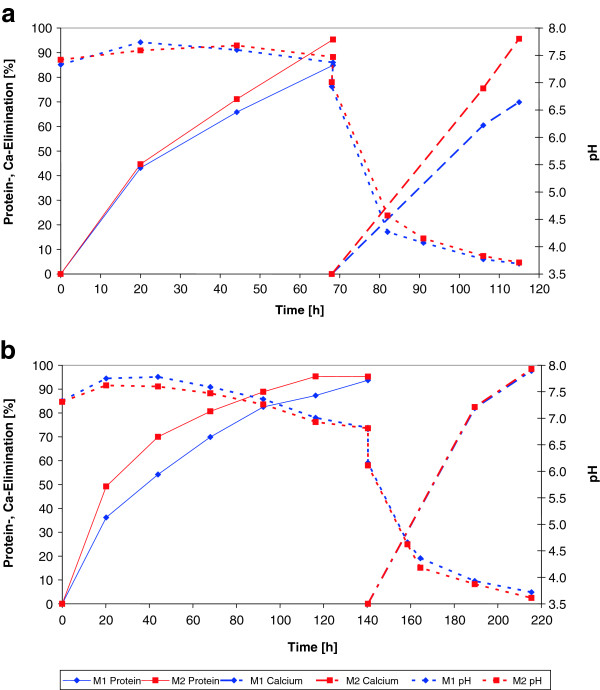
**Deproteination of a) wet shrimp shells and b) dried shrimp shells by enrichment cultures M1 and M2.** Decalcification with lactic acid, produced *in-situ* by lactic acid bacteria (LAB) from glucose was started after 68 h in the wet shrimp assay **(a)** and after 140 h in the dried shrimp assay **(b)** by addition of glucose and LAB. Twice the amount of glucose had to be added in the dry shrimp shell assay as compared to the assay with wet shrimp shells, due to the higher amount of total solids in dry shrimp shells.

To elucidate whether there exist relevant differences for deproteination of wet or dried *P. monodon* shells, protein and calcium elimination from the respective abdomen fractions with the proteolytic enrichment cultures M1 and M2 were compared (Figure 1a and b). TKN and protein content of waste shells were determined before each assay. The average TKN and protein content of the shells were 73 mg/g wet weight and 300 mg/g dry weight, respectively as described previously [2]. As shown in Figure 1a, 95% of the protein of 15 g wet weight of shrimp shells (=5.1 g dry weight) was eliminated after 68 h of incubation with enrichment culture M2 and more than 80% was removed with enrichment culture M1. About 80% of the dissolved protein was deaminated during deproteination of wet and dried shrimp shells by both enrichment cultures (Table 1). Although protein removal from dried shrimp shells (Figure 1b) required 120 h (culture M2) or 140 h (culture M1), yet the protein removal rates were much higher than with wet shrimp shells if the dry matter content is taken in consideration. The dry matter amount was 14.1 g and 5.1 g per assay of dried and wet shrimp shells, respectively (Table 2).

**Table 1:** Increase of the percentage of ammonia in soluble total Kjeldahl Nitrogen (TKN) with time

**Time period (h)**	**Wet shell assay M1 (% ammonia)**	**Wet shell assay M2 (% ammonia)**	**Dry shell assay M1 (% ammonia)**	**Dry shell assay M2 (% ammonia)**
0	0	0	0	0
22	57	59	61	61
44	65	76	67	69
68	70	80	72	75
92	75	83	77	80

**Table 2:** Comparison of nitrogen-elimination rates of shrimp shells by enrichment cultures M1 and M2

**Time period (h)**	**Wet shell assay M1 (mg N/h) 5.1 g dry weight**	**Wet shell assay M2 (mg N/h) 5.1 g dry weight**	**Dry shell assay M1 (mg N/h) 14.1 g dry weight**	**Dry shell assay M2 (mg N/h) 14.1 g dry weight**
0-20	0.35	0.36	0.80	1.08
20-48	0.15	0.18	0.33	0.38
48-72	0.13	0.16	0.29	0.24
72-96	n.d.	n.d.	0.23	0.18
96-120	n.d.	n.d.	0.09	0.12

The total mass addition to assays with wet and dried shrimp shells was 15 g, resulting in different dry weight amounts.

After deproteination of the shrimp shells, calcium compounds are still incrusted in shells and are responsible for the mechanical stability. To obtain pure chitin, a smooth dissolution of calcium compounds in shrimp shells is possible with lactic acid that forms complexes with Ca^2+^ ions. Since lactic acid as such is expensive, *in-situ* production by lactic acid bacteria LAB from carbohydrates seems to be an elegant method for calcium removal [7-9]. In comparison to chemical treatment with alkali and mineral acids for protein and calcium removal, respectively, biological removal of these compounds proceeds under much less extreme conditions, but requires a longer incubation time [1,2,7,9]. In our study, demineralization of wet or dried shrimp shells was started after deproteination at 68 h or 140 h, respectively, by addition of LAB from a bio yoghurt and glucose as a carbon source (Figures 1a and b). The pH dropped during lactic acid formation from glucose and decalcification apparently proceeded to completion when enough lactic acid for complex formation with Ca^2+^ ions was generated by the LAB (Figure 1, M1 and M2). When calcium was not completely removed from shrimp shells by the first glucose feeding, glucose was re-fed, which led to more lactic acid production and resulted in completion of calcium solubilisation. Alternatively, the acid supernatant was replaced with fresh medium that contained glucose and LAB for more lactic acid production and calcium solubilisation (data not shown). Finally almost 100% calcium was released by the LAB from bio yoghurt.

In a parallel test, it was found that the protein elimination rates were much higher and the time requirement for deproteination of shrimp shell chitin was much shorter with the enrichment cultures M1 and especially M2 as compared to the autochthonic population on the shells alone (data not shown). This might have been due to the high number of proteolytic and peptolytic bacteria in the inoculum that were enriched with peptone-containing media. Besides enrichment cultures M1 and M2, a mixed bacterial population (data not shown) was also enriched in media m1 and m2 from minced meat, which did not express any chitinase activity during deproteination, that otherwise would have lowered the quality of the chitin. Such enrichment cultures might be applied for deproteination of shrimp shells in a non-sterile environment. Under practical conditions after an incubation time of more than a week, significant growth of chitin-degrading bacteria in parallel to chitinase-negative bacteria must be considered. Low ammonia concentrations in the deproteination solution indicated little deamination activity which makes the solution of dissolved proteins and peptides suitable as animal feed additives. As shown in Table 1, deamination unfortunately started immediately and 75–83% of the soluble TKN were deaminated within 92 h, no matter whether wet or dried shells were used. Most of the ammonia was produced during the first 22 h of incubation, indicating that proteolysis and deamination proceeded in parallel.

### Comparison of viscosity of chitin prepared by different methods

**Figure 2:**
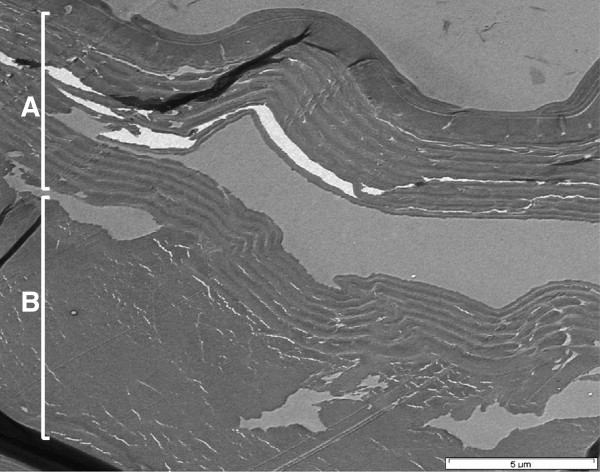
**Structure of chitin after chemical treatment of shrimp shells.** Disruption and dissolution of the epicuticle and damaged layers of the exocuticle (layer A) and endocuticle (layer B) are visible in the micrograph.

**Figure 3:**
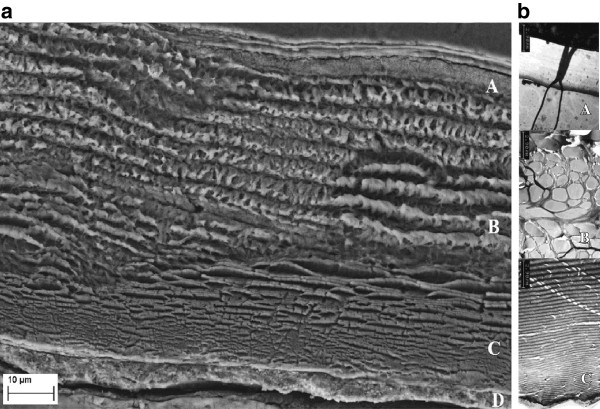
**SEM and TEM of untreated waste shrimp shells. a)** SEM micrograph of a thin section and **b)** TEM micrograph of different regions of a *P. monodon* shrimp-shell. **A** = Epicuticle **(**with a channel in Figure 3**b)**, **B** = exocuticle, **C** = endocuticle and **D** = inner membrane of shrimp shells.

Viscosity is an important factor which indicates desired properties of chitin or chitosan. It is one of the major quality criteria for industrial production of chitosan and its applications. For this reason, the viscosity of chitin that was obtained by biological deproteination and decalcification was compared with chitin which was prepared by sodium hydroxide hydrolysis of proteins and subsequently, calcium was removed by hydrochloric acid. Chitin from chemical protein hydrolysis and decalcification had a much higher viscosity than the chitin that was prepared by bacterial deproteination and demineralization. Beaney et al. [9] also found that the quality of chemically processed chitin was better than the chitin processed with lactic acid fermentation from the shells of *Nephrops norvegicus.* In the present study, the chemically and biologically processed chitin batches were compared with a 99% pure chitin purchased from a chemicals company. The commercially available chitin had the lowest viscosity (Table 3). Therefore, a linear relationship between the chitin purity and viscosity may be ruled out and as evident from the results, protein and mineral contents are not the only parameters to be considered for the chitin quality. In our previous study [1], viscosities of chitin and chitosan extracted from shrimp shells were highly dependent on the method of preparation, even when the degree of deproteination and decalcification were similar in the end product. The SEM micrograph of chemically prepared chitin (Figure 2) clearly indicates that the shell cuticles were not intact anymore after harsh chemical treatment when compared to untreated shells (Figure 3A). Bade et al. [10] reported that after acid hydrolysis, the chitin structure no longer retained its native fine fibrous and covalently stabilized form, and it lost its resistance to chitinase activity of autochthonic bacteria. Structural disaggregation of chitin during defined chemical or biological purification might have led to a loosely packed chitin which could be dissolved in N-methyl-2-pyrrolidone (NMP)/LiCl solution and resulted in a liquid with a high viscosity. Although the commercially available chitin had the highest purity, it was least soluble in N-methyl-2-pyrrolidone (NMP)/LiCl solution and had the lowest viscosity. Molecular weight distribution after purification and the grade of polymerisation are apparently main factors that determine the viscosity of chitin. Different batches of biologically processed chitin from *Penaeus monodon* had molecular weights ranging from 0.3 - 1.8 MDa. Constant viscosities of chitin or chitosan solutions are required for application in the food and pharma industry [1,4] or for supply of chitin/gelatin membranes for tissue engineering [11].

**Table 3:** Viscosity of chitin from shrimp shells, purified chemically or biologically

**Sample**	**Viscosity (mPa.s)**	**Purity (%)**
Chitin prepared chemically	300	>98
Chitin prepared biologically	45-135	93-94
Commercial chitin	28	99

### Structural and chemical characterization by electron microscopy

To obtain information about the structure of shrimp shells of *Penaeus monodon,* thin sections of samples were prepared for microscopy. Since shrimp shells tended to fold up when prepared for TEM inspection, polished surfaces of bulk resin samples with embedded shrimp shells were additionally investigated by SEM as well as with light microscopy. The epicuticle, exocuticle, endocuticle and inner membrane were visible as distinct layers in micrographs of a thin section of the abdomen of *P. monodon* (Figure 3) which were similar to the layered cuticle structure of *Carcinus maenas* as described by Roer and Dillaman [6]. The shell structure appears to be fibrous and the purified chitin may or may not retain this fibrous character after processing of shells, depending upon the method of purification [10,12]. In our study, the thickness of the epicuticle was approximately 3.2 μm, that of the exocuticle was about 33.5 μm and that of the endocuticle was about 16.1 μm, as estimated from the SEM micrograph of waste shrimp shell samples (Figure 3b).

**Figure 4:**
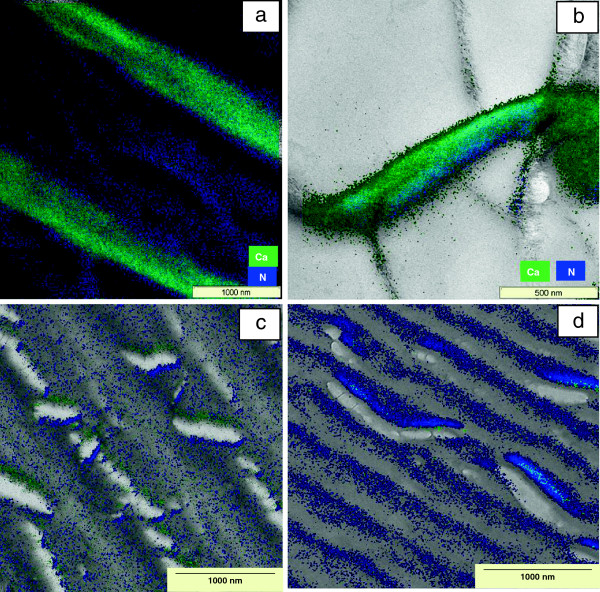
**TEM based structural and elemental investigations of shrimp shells.** Comparison of morphological details of *P. monodon* shrimp shells with element distribution by overlay of bright-field and energy-filtered TEM images in **a)** Exocuticle and **b)** Endocuticle before biological chitin treatment, **c)** Endocuticle between deproteination and demineralization and **d)** Endocuticle after deproteination and demineralization.

**Figure 5:**
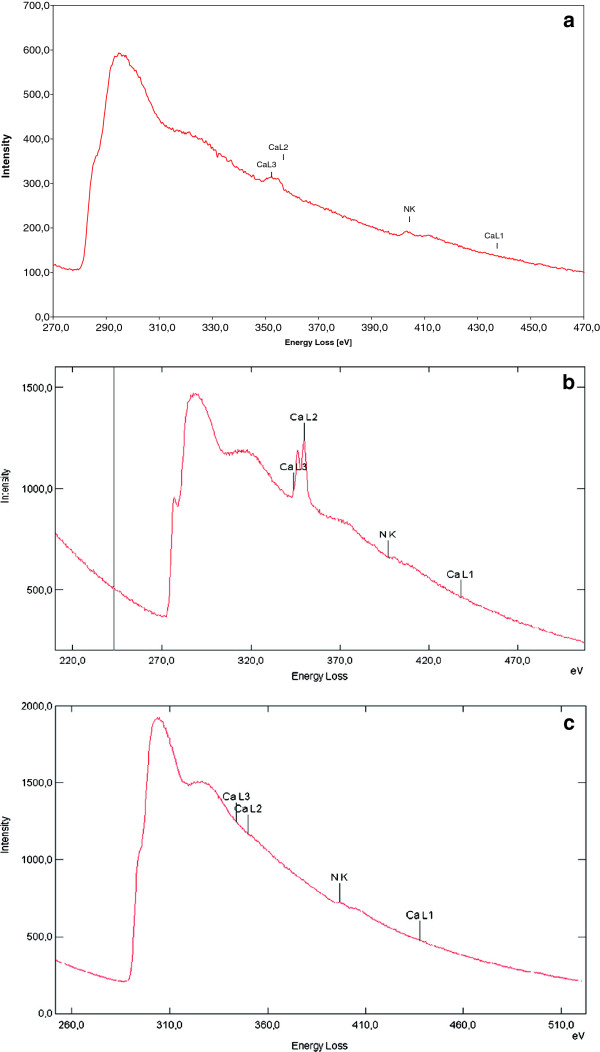
**EEL spectra of shrimp shells during different stages of biological chitin purification. a)** Exocuticle of untreated *P. monodon* shrimp shells, **b)** Endocuticle of *P. monodon* shrimp shells after deproteination when protein (nitrogen) was largely removed and **c)** Shrimp shells after demineralization when calcium was removed. CaL1, CaL2, CaL3 and NK represent energy loss intensities from electron shells of respective elements.

The combination of imaging and analytical techniques of TEM allowed the correlation of morphological details with their micro chemical properties. TEM examination combined with techniques such as electron energy loss spectroscopy (EELS) could give information about the location of nitrogen and calcium in the shells. With this method, nitrogen in the samples was investigated that acted as a marker for the distribution of protein before, during and after deproteination. Nitrogen is an element present in both, chitin and protein structures. By studying various phases during deproteination of shells, chitin layers can be located by a constant N-content. After deproteination most proteins are solubilised and most amino acids are deaminated. Similarly, the calcium content before and after demineralization in different regions of the shells could be identified. These elements were localized two-dimensionally by energy-filtered TEM through comparison of element-specific maps with bright-field TEM images. In order to elucidate the influence of deproteination and demineralization on the morphology and chemical composition of the shrimp shells, non-processed shrimp shells were taken as the reference to represent the initial chitin status when no biological or chemical treatment was applied to the shells. For a better visualization of structural and chemical peculiarities, the TEM bright-field images were compared with the element maps by an overlay presentation (Figure 4). As visible in the color-coded map, nitrogen as well as calcium was present in the exocuticle (Figure 4a). Similar findings were obtained for the endocuticle (Figure 4b). After deproteination of wet shrimp shells with enrichment cultures of proteolytic bacteria, structural damage of the layers in the endocuticle region could be deducted from Figure 4c and d. This could also explain why a portion of calcium was already released through deproteination. This calcium was a former part of the exocuticle. Since a non-negligible amount of nitrogen was found in regions of the exocuticle, it could be assumed that this nitrogen belonged to chitin. Demineralization of the shrimp shells after incubation with LAB could be observed by EELS. In comparison to the EEL spectra of untreated or deproteinated shrimp shells (Figure 5a and b), the Ca-L_2_ and Ca-L_3_ signals were nearly missing in demineralised shells (Figure 5c). The energy-filtered TEM images of the Ca distribution (cf. Figure 4) recorded in the area of the endocuticle revealed only traces of this element. However, nitrogen was found in extended regions at relatively high concentration in the endocuticle, which would have been incorporated in chitin and protein as well. Thus, the N in EEL spectra of purified shrimp shells after demineralization represents the N-content of chitin. The protein between chitin layers may not be dissolvable by short term deproteination and thus protein could not be eliminated entirely. Decalcification with lactic acid may however release most of the residual proteins. It has been reported that lactic acid production and the resulting pH drop during fermentation of shrimp wastes with LAB activates proteases [8]. This is why in the chitin, protein may have been removed completely, leaving behind only the N-content of the N-acetyl-glucosamine of chitin (Figure 5). By mass-balances, it was obvious that the weight loss between deproteinated chitin fractions and chitin after subsequent decalcification was not entirely due to calcium elimination (data not shown).

### Evaluation of protein impurities by solid state ^13^C cross polarization/magic angle spinning nuclear magnetic resonance (CP/MAS NMR) spectroscopy

**Figure 6:**
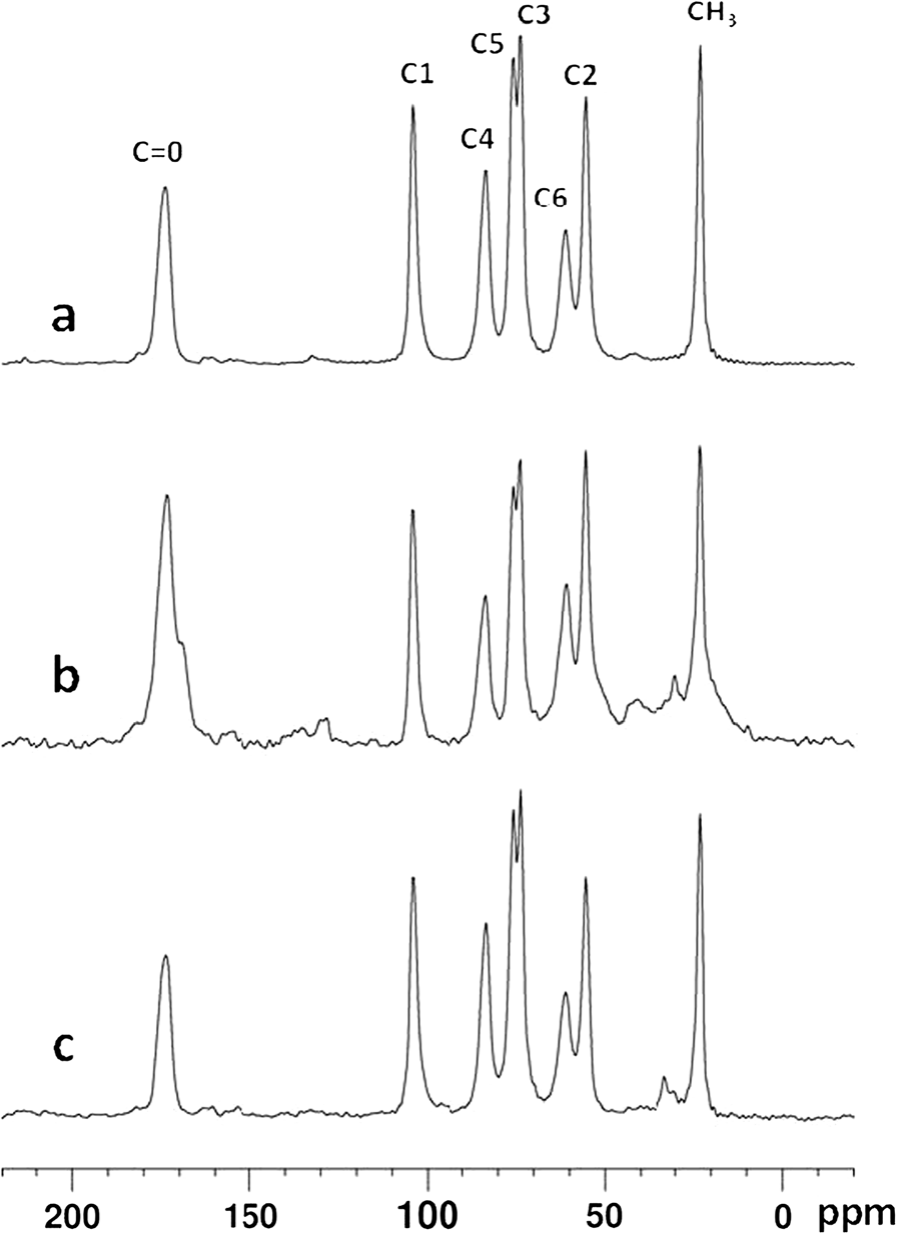
^**13**^**C CP/MAS NMR spectra of shrimp shell and different chitin.** The spectra were measured under 10 kHz MAS spinning at a ^13^C resonance frequency of 75 MHz. **a)** Biologically purified chitin, **b)** Shrimp shells before biological treatment and **c)** Commercial chitin. The signal at 48 ppm is a spinning sideband of the carbonyl signal at 175 ppm. The weak signals in the regions of 20–40 ppm, near 120 ppm and the up field shoulder of the carbonyl peak at 175 ppm in Figure 6b are attributed to aliphatic, aromatic and carbonyl resonances of residual protein contaminations, respectively.

Chitin is a polymer of β-(1–4)-N-acetyl-D-glucosamine (NAG) units [1]. Single lines of individual carbons could easily be obtained in chitin spectra of a solid state NMR without the need of solubilizing this polymer in a solvent [13,14]. The characteristic ^13^C chemical shifts of sugars and protein allows an analysis of the chemical composition of the shrimp shells, provided high resolution can be achieved. For this aim, magic angle spinning was used to reduce the wide line spectra of solid chitin material to their isotropic signals, resulting in resolved ^13^C CP/MAS spectra (Figure 6a and b). The spectra of shrimp shells with and without biological treatment were compared with the NMR spectrum of a commercially available chitin as a reference (Figure 6c). Eight signals were observed in all cases, which have been previously assigned to the 8 carbon sites of chitin [15,16]. Only the spectrum obtained from the sample of commercially distributed chitin displayed some minor additional signals near 30 ppm lying in the aliphatic region. Signals from compounds other than chitin might be obscured by the chitin spectrum, if their chemical shifts coincided with those of chitin. For example, the carbonyl signal of chitin near 175 ppm would overlay the respective signal of protein material. However, as a substantial contribution of protein material arises from aliphatic e.g. C beta, C gamma carbons in the region around 20–40 ppm, any carbonyl signal from proteins would be accompanied by resonances in this frequency region as well. The CH_3_ groups in biologically treated samples produced signals of almost the same intensity as in untreated shrimp chitin and in purchased chitin (Figure 6) indicating a similar degree of acetylation in all three samples with none or minimal deactylation during chitin purification from shrimp shells. The observations in this study are comparable with those of Teng et al. [3] who found C-1 to C-6 carbons of the main *N*-acetyl glucosamine between 50 and 100 ppm, the carbonyl peak (acetyl group) around 170 ppm and the methyl peak of the acetyl group around 20 ppm in the different chitin samples that were prepared during concurrent fermentation of shrimp shells with three proteolytic strains of *Aspergillus niger*.

As no extra signal was observed within the limits given by the signal/noise ratio (S/N) in the treated shrimp shell samples, and only a minor signal of less than 3% was observed in the commercial chitin, impurities can only constitute a negligible part of the sample. From the S/N ratio and by considering the unlikely possibility that all signals of impurities were coinciding with the chitin spectrum, a conservative upper limit for impurities of around 3% was estimated for the treated shrimp shells. This indicated that the final product might have contained more than 97% chitin and, thus, the biological chitin extraction process used in this study has achieved a clean chitin. The quality of the obtained chitin was much better than that of the commercially available chitin used as a reference.

## Conclusions

The proteolytic culture M2 was the more efficient of the two enrichment cultures that were used for deproteination of both wet and dried shrimp shells. Chitin purification from wet shrimp shells was apparently faster but considering the solid content, deproteination rates of dried shrimp shells were 3 times higher than those of the wet shrimp shells. Therefore, it is important to consider both, wet and dry weight load for plant designing purposes. Although the viscosity of chemically extracted chitin from wet shrimp shells was higher than that of biologically extracted chitin, the biological method may be used as an environmentally friendly chitin production alternative for low cost applications, where highly viscous chitin is not the prime requirement. The biologically purified chitin was much better than the chemically purified, commercially available chitin but was not as good as the chitin obtained by an optimized chemical purification procedure, developed in our laboratory previously [1].

With the accompanying EM investigations during various phases of chitin extraction, precise information about the location of chitin and protein could be gained. The scanning of shells and the intermediate products during chitin extraction indicated whether the nitrogen as a part of protein or as a part of chitin was eliminated during shell digestion by bacteria. After deproteination, the structure of both, epicuticle and exocuticle was changed, indicating that most protein was located here. Since the epicuticle is relatively thin, the abundant amount of proteins must be located in the exocuticle. This may be the reason why more than 30% of the calcium was removed during deproteination, but could not be measured as solubilized calcium. The calcium of the exocuticle was eliminated in a colloidal form and removed with the protein solution. By NMR analysis it was found that the final product contained more than 97% chitin and less than 3% impurities. This shows that the biological chitin extraction process used in this study has achieved a clean chitin.

## Materials and methods

### Chitin purification from shrimp shells

A) Biological method: For chitin purification, moist *Penaeus monodon* shrimp shells from Indonesia were taken as such or after air-drying for two days under a laboratory laminar air flow hood. The autochthonic population of wet shrimp shells served as an inoculum to generate chitinase-free enrichment cultures for microbial deproteination in the media as mentioned later. For biological decalcification, lactic acid bacteria (LAB) were revived from a commercially available bio-yoghurt.

B) Chemical method: Wet shrimp shells were soaked in 10% HCl (1:4) at 23 ± 2°C with constant stirring on a magnetic stirrer. After 1 hour of incubation, the shells were sieved and thoroughly washed with tap water. Draining of excessive water was done manually with a potato press. The moist demineralised shells were then subjected to deproteination by incubation with 2 M NaOH (shell/alkali ratio = 1:6, w/w) at 50 ± 5°C for 2.5 hours on a rotating shaker. After cooling for 15–20 minutes, residual chitin was sieved off and washed with tap water until a neutral pH of the spent water. The end product was free of minerals and contained ≤1.8% protein.

### Preparation of enrichment cultures

Mixed cultures M1 and M2 of autochthonic bacteria from shrimp shells were enriched in peptone containing media m1 and m2. Medium m1 contained per liter: 5 g peptone from soy beans, 15 g peptone from casein, 5 g NaCl, 6.81 g KH_2_PO_4_, 11.4 g K_2_HPO_4_, adjusted to pH 7. Medium m2 contained per liter: 6 g peptone from meat, 3 g peptone from casein, 5.2 g sodium caseinate, 5 g NaCl, 0.2 g KCl^.^2H_2_O, 0.32 g Na_3_C_6_H_5_O_7_, 0.04 g KH_2_PO_4_, 0.13 g K_2_HPO_4_, adjusted to pH 7. Typically, 20 ml portions of media in serum bottles were inoculated with shrimp shells for enrichment of proteolytic bacteria M1 and M2. After inoculation serum bottles were closed with rubber stoppers and the gas phase was exchanged with nitrogen (Linde AG, Pullach, Germany) at a gas station. Incubation was done at 37°C under gentle shaking (Lab-Shaker model Kühner, B.Braun, Melsungen, Germany). After several transfers of culture supernatant into fresh medium, chitinolytic bacteria in both enrichment cultures M1 and M2 were expected to be washed out, but molecular tests still revealed the presence of chitinase genes in both cultures when compared with homologous chitinase genes in the DNA of chitinase-positive *Serratia marcescens*[17]. Since no structural changes of chitin occurred within 5 days of incubation with these cultures and 3 or 6 days were required for deproteination of wet or dried shrimp shells, respectively, these enrichment cultures were selected for the present study. For deproteination experiments, 250 ml cultures were grown in 500 ml Schott flasks that contained 250 ml medium m1 or m2.

### Deproteination and demineralization of shrimp shells

Deproteination of shrimp shells was performed with 250 ml of proteolytic bacteria of enrichment cultures M1 or M2. The bacteria were harvested by centrifugation (Model 5403, Eppendorf, Hamburg, Germany) at 7000 g for 12 minutes, the supernatant was discarded and the wet weight of the cell pellet was determined. Then the pellet was re-suspended in 250 ml of tap water and 15 g wet (moisture content about 66%) or dried (moisture content about 6%) shrimp shells were added. In a parallel test with m1 medium and tap water, it was found that tap water was also suitable for bacterial growth and all required nutrients for bacterial growth could be obtained from the shrimp shells itself. To obtain anaerobic conditions, air in the gas head of the closed bottles was exchanged with nitrogen and the bottles were incubated on a rotary shaker at 37°C and 110 rotations per minute. During incubation samples were taken at regular intervals to measure the dissolved total N, NH_4_^+^, TKN, pH and dissolved Ca concentrations with the methods as described previously [1,2]. The protein content of the solid deproteinated chitin was measured by subtracting TKN of chitin from TKN of the untreated sample and multiplying it with 6.25 as described in our previous study [2]. The soluble protein was measured as described by Bradford [18].

After complete deproteination, the calcium-containing shrimp shells were filtered off with a nylon bag and were washed several times with tap water. In the end a “lime-chitin” was obtained which was air-dried under an exhaust cabin.

For demineralization, 250 ml MRS medium [19] was inoculated with 10 ml bio-yoghurt, which contained live lactic acid bacteria (LAB). After 24 h of growth, 10% of the suspension was transferred into 250 ml of fresh MRS medium and incubated for another 24 h at 37°C on a rotary shaker at 110 rotations per minute. Lactic acid bacteria were then harvested by centrifugation and used for decalcification of deproteinated shrimp shells. The LAB (around 3 g wet weight) were resuspended in 250 ml of tap water that contained 20 g/l glucose and then the suspension was added to the “lime-chitin” from the deproteination assay. The air in bottles was replaced with nitrogen and the assay was incubated at 37°C on a rotary shaker at 110 rotations per minute.

Due to lactic acid formation from glucose during incubation, a drop of the pH was observed and calcium was solubilised (Figure 1a, 68 h or Figure 2b, 140 h onwards). After demineralization the chitin was transferred into a nylon bag, washed several times with distilled water and pressed manually to squeeze out most of the moisture before air drying.

### Measurement of chitin viscosity

The viscosity of different chitin samples was determined with a Brookfield viscosimeter RVDVII CP (Bruchsal, Germany) by solubilising 0.2 g of chitin in 100 ml of N-methyl-2-pyrrolidone (NMP)/LiCl (5%) solution with constant stirring for 10–12 hours at 23 ± 2°C. The measurements were made at a shear rate of 56.3/s in a temperature controlled steel cone by maintaining 20°C. The sample volume was 0.5 ml. Chitin purchased from Sigma, Germany was used as reference. The viscosity-average molecular weight of chitin was calculated by the method of Hoffmann et al. [20].

### Preparation of samples for microscopy

For microscopic characterization 2 mm pieces of the *P. monodon* shells were fixed with 2.5% glutaraldehyde in 0.1 M cacodylate buffer at a pH of 7.4 at room temperature [21]. After incubation for 1 h the shrimp shells were washed with 0.1 M cacodlyate buffer until the smell of glutaraldehyde disappeared. After washing, the sample was dehydrated via an ascending series of suspensions in 70%, 90% and 2 × 100% ethanol. The water-free samples were then transferred into the embedding medium London Resin (LR) White, where the concentration of embedding agent was successively increased step-wise. Subsequently, polymerization was carried out at 60°C under exclusion of atmospheric oxygen for 24 h.

### Light microscopy

For inspection of the shrimp shells morphology, 5 μm thin sections were prepared by using an ultra cut microtome (Leica, Germany) equipped with a diamond knife (Diatome AG, Biel, Switzerland). The cuts were transferred onto glass slides that were coated with chromium gelatine (0.1% gelatine and 0.01% CrK (SO_4_)_2_ · 12H_2_O). Then a selective staining with 0.1% (w/v) toluidine blue in 2.5% aqueous sodium carbonate solution was carried out as described by Trump et al. [22]. The stained shrimp shell cross sections were then embedded in Entellan^®^ and observed under a Diaplan light microscope (Leitz, Germany) equipped with a Leica DFC 500 camera.

### Scanning electron microscopy (SEM)

In order to perform SEM, shrimp shells were crushed before embedding in a synthetic resin. Then, thin sections of about 70 μm thickness were generated with an ultra microtome as described above. To avoid charging of the non-conducting samples under the electron beam, thin sections were coated with a few nm thick layer of carbon by evaporation deposition. The structure of the polished shrimp-shell sections was imaged by means of secondary electrons (SE) in a scanning electron microscope of the type LEO 1530 Gemini (Carl Zeiss NTS GmbH, Germany) at 10 kV accelerating voltage. This microscope was equipped with a thermally assisted field emission gun. SE signals were gathered by a conventional Everhart-Thornley detector.

### Transmission electron microscopy (TEM)

For TEM, 60–90 nm thin sections of the shrimp shells were prepared with the ultra microtome as described above. Copper grids (100 mesh) covered with 1.4% Pioloform (polyvinyl butyral) films were used as sample carriers. To increase their contrast they were stained for 5 min in 8 mmol/l lead citrate solution to exclude air-borne CO_2_[23]. Samples were characterized by using a Zeiss EM 912 Omega microscope with a thermal lanthanum hexaboride cathode at an acceleration voltage of 120 kV. Information about the sample morphology was obtained by mass-thickness contrast in the conventional TEM mode with a 1 K CCD digital camera (Proscan, Germany). Overview images were taken by means of electronic film plates (Ditabis, Germany). Since the microscope was equipped with an imaging energy filter of the Omega type (Zeiss Leo 912), four magnetic prisms were arranged between the diffraction lens and the first projector. Chemical analyses of nm regions were performed by electron energy loss spectroscopy (EELS) and two-dimensional mapping of the element distribution within the transmitted volume by means of energy-filtered TEM (EFTEM). In order to obtain element-specific maps, both the three-window method and the two-window ratio technique were applied [24,25]. In particular, the lateral distribution of nitrogen and calcium was imaged by the use of N-K ionization edge (threshold energy at approximately 402 eV) and the Ca-L edge (about 346 eV), respectively. For energy-filtered TEM a slit width of about 25 eV and measuring times from 10 s to 40 s were used.

### Solid state nuclear magnetic resonance (NMR) spectroscopy

To perform NMR, about 10 mg of dry chitin shells were ground and filled into a 4 mm outer diameter ZnO_2_ rotor. Solid state ^13^C NMR spectra of the chitin samples were acquired under magic angle spinning at a spinning frequency of 10 kHz by using a Bruker DMX spectrometer at a ^13^C resonance frequency of 75 MHz (corresponding to a ^1^H resonance frequency of 300 MHz), a cross polarization sequence employing a B_1_ field strength of 50 kHz with ramp 80 – 100% on the ^1^H channel and a contact time of 5 msec. The signal was acquired for 20 msec under simultaneous hetero nuclear ^1^H decoupling of 80 kHz. A rotor synchronized echo was used to avoid baseline artefacts. Approximately 1000 scans were averaged, with a recycling time of 10 sec between successive scans. Spectra were processed with 10 Hz line broadening.

## Abbreviations

CP/MAS: Cross polarization/magic angle spinning; EELS: Electron energy loss spectroscopy; EFTEM: Energy-filtered transmission electron microscopy; LAB: Lactic acid bacteria; m1 and m2: Growth medium 1 and growth medium 2; M1 and M2: Enrichment culture 1 and enrichment culture 2; NMR: Nuclear magnetic resonance; SEM: Scanning electron microscopy; TEM: Transmission electron microscopy; TKN: Total kjeldahl nitrogen.

## Competing interests

The authors declare that they have no competing interests.

## Authors’ contributions

CG and JW conceived the research idea, provided supervision to YX by intensive discussions during lab work and contributed to the preparation of this manuscript. YX carried out the laboratory work for enrichment of microbial cultures and biological chitin purification. MB contributed results on chemical chitin purification, carried out viscosity studies, interpreted results and wrote the final draft of this manuscript. RS contributed his expertise in electron microscopy investigations. SG and AU carried out the NMR studies and interpreted NMR data. All authors read and approve the final manuscript.
